# Effects of topiramate, bupropion and naltrexone isolated or combined on subcutaneous adipose tissue in obese rats

**DOI:** 10.31744/einstein_journal/2022AO5587

**Published:** 2022-05-16

**Authors:** Monica Alhadas Scudeler, Stephania Morreale, Lorena Doretto-Silva, Giuliana Petri, José Francisco Ramos dos Santos, Cristina Nassis, Olga Maria de Toledo Correa, Juliana Mora Veridiano

**Affiliations:** 1 Centro Universitário FMABC Santo André SP Brazil Centro Universitário FMABC, Santo André, SP, Brazil.

**Keywords:** Topiramate, Bupropion, Naltrexone, Obesity, Subcutaneous fat, Body weight, Rats

## Abstract

**Objective:**

To evaluate the effects of combining topiramate, bupropion and naltrexone in obesity-induced rats on their weight and subcutaneous adipose tissue.

**Methods:**

A total of 40 male Wistar rats were induced to obesity for 8 weeks and the animals were divided into 8 groups: Ctr - control, G0 - Sham, G1 - oral saline solution (1.0mL/day), G2 - topiramate (20.0mg/kg) and bupropion (5.0mg/kg), G3 - naltrexone (20.0mg/kg), G4 - topiramate (20.0mg/kg), G5 - bupropion (5.0mg/kg) and G6 - topiramate (20.0mg/kg), bupropion (5.0mg/kg) and naltrexone (20.0mg/kg). During the experiment, all animals were weighed weekly. After 30 days of treatment animals were euthanized and their skin fragments were processed and stained with hematoxylin and eosin for morphological, morphometric and biochemical analyzes.

**Results:**

The only group that presented a decrease in the volume of subcutaneous adipose tissue was G3, but this decrease was not significant when compared with the other groups. The G4, the G5 and the G6 presented increased adipose tissue volume. Data showed that until the eighth week all animals increased their weight by approximately 50%. After treatment animals of all groups, except G3, increased their weight from 4% to 9% approximately. The G3 was the only group that lost weight, but this decrease was not significant.

**Conclusion:**

The medicines studied were not efficient in reducing weight in obese rats. However, it should be considered that 30-day treatment period is not enough to observe the stronger effects of these drugs.

## INTRODUCTION

Obesity is a body condition characterized by the excess of adipose tissue in the body. This is a disease resulting from a nutritional imbalance caused by a positive energy balance, *i.e.*, caloric intake is greater than the caloric output capacity.^(1)^

This disease is caused by multifactorial disorders and depends on the interaction of genetic, metabolic, social, cultural, and behavioral factors.^(2)^ Among these factors observed in obese individuals, psychological issues such as depression, anxiety, stress, and binge eating disorder are highlighted.^(3-5)^

Treatment for obesity requires nutritional counseling, cognitive behavioral psychotherapy, and the use of drugs, which can be of 3 different classes: antidepressants, appetite suppressants acting on the central nervous system, and anticonvulsants.^(6)^

Topiramate, bupropion and naltrexone are the new drugs under study for treatment of eating disorders, and consequently, to treat obesity with promising clinical results.^(7)^

Bupropion is characterized as an antidepressant that inhibits the reuptake of dopamine and noradrenaline. Increased levels of these 2 catecholamines stimulate the activity of pro-opiomelanocortin (POMC) producing neurons, located in the arcuate nucleus in the hypothalamus. The POMC consists of a precursor polypeptide and its cleavage gives rise to, among other products, ∂-melanocyte-stimulating hormone (∂-MSH) and ß-endorphin, an endogenous opioid. Increased ∂-MSH promotes activation of melanocortin 4 receptor (MC4R) and the secondary signaling pathways coupled to them, leading to increased energy expenditure, and decreased appetite. The drug has the effect of reducing anxiety symptoms and the frequency of compulsive episodes, suggesting an effect on weight reduction, and improved sexual performance.^(8)^

Topiramate is an anticonvulsant drug considered a promising medication in the treatment of obesity and binge eating disorder (BED). Studies in obese populations have demonstrated a significant reduction in binge eating episodes and subsequent weight loss.^(9)^

In addition, topiramate is currently used in the treatment of epilepsy and migraine, as it is an antagonist of the amino-3-hydroxy-5-methyl-isoxazol-4-propionic acid and kainate (AMPA/KA) receptor. This medication is also responsible to increase gamma-aminobutyric acid (GABA) activity at GABA receptors by triggering stabilization of sodium and calcium channels. Topiramate has a GABAergic mechanism of action, but it is also an anorexigen, and currently it is used successfully in treatment for weight loss.

In animal models, topiramate has been shown to reduce appetite as well as to interfere with the efficiency of energy using, being the latter a consequence of increased thermogenesis and fat oxidation (due to stimulation of lipoprotein lipase in brown adipose tissue and skeletal muscle), which leads to weight loss. When topiramate was tested in obese patients with BED, good results were observed in weight loss and reduction of BED scores.^(10)^

Naltrexone is an opioid receptor antagonist, initially approved as a treatment for opioid dependence and later for alcohol dependence.^(11)^ Some studies in rats have shown that ingestion of palatable foods (*e.g.*, sweets) leads to increased levels of β-endorphin in the hypothalamus. This finding led to the opioid-mediated palatability hypothesis, which assumes that opioid release has an expression on food palatability and vice versa. Some anecdotal reports from humans in the 1980s suggest that naltrexone, while having no direct effect on hunger, would lead to a decrease in the good feeling after eating, which would result in reduction of food intake.^(12)^

Although pharmacotherapy is widely used in clinical medicine for the treatment of obesity, there is no scientific evidence of the effect of these drugs on weight loss. This study aims to clarify the action of these medicines.

## OBJECTIVE

To evaluate the effects of the association of topiramate, bupropion and naltrexone in weight and subcutaneous adipose tissue in rats induced to obesity.

## METHODS

The study was conducted at *Faculdade de Medicina do ABC*, Santo André, Brazil, from August 2017 to December 2017. A total of 40 male Wistar rats aged 14 to 16 weeks, weighing between 150-250g, were included in the study. Animals provided by the Animal House of the *Faculdade de Medicina do ABC* were kept in individual cages. During treatment period animals were fed with water and Nuvital CR-1 ad libitum. The light-dark cycle was also controlled and established as 12 hours in each phase. The humidity of the vivarium was maintained between 60% and 80%, and the environment temperature was ≈22˚C. This study was approved by the Ethics Committee on Animal Use, number 03/2017.

Thirty-five animals were subjected to the ingestion of a highly palatable food with high sugar and fat content (Nestle^®^ Moça Sweetened Condensed Milk) for 8 weeks and were received by using the gavage method. The ingestion of a food with high sugar and fat content was necessary as an attempt to develop compulsive eating process and consequent induction of obesity in the animals.

The ratio of normal food and highly palatable food was 1:1, *i.e.*, half feed and half condensed milk at 5g/100mg body weight. All animals were weighed weekly. Five rats were maintained without intake of highly palatable food, and they received the normal diet only. Animals not subjected to any medicine treatment were used as Control Group (Ctr).

Animals submitted to the intake of the highly palatable food were divided into 7 groups with 5 rats each: Group 0 (G0/sham) - animals that were not submitted to treatment, Group 1 (G1) - those that received saline solution orally (1.0mL/day), Group 2 (G2) - those that received topiramate (20.0mg/kg) and bupropion (5.0mg/kg), Group 3 (G3) - those that received naltrexone (20.0mg/kg), Group 4 (G4) - those that received topiramate (20.0mg/kg), Group 5 (G5) - those that received bupropion (5.0mg/kg), Group 6 (G6) - those that received topiramate (20.0mg/kg), naltrexone (20.0mg/kg) and bupropion (5.0mg/kg). Animals were submitted to the treatment for 30 consecutive days.

Drugs used were solubilized in saline solution to reach a concentration of 1mL/day for each animal. The reference commercial forms of the drugs used were: topiramate (Topamax^®^, Janssen-Cilag Farmacêutica Ltda., São Paulo, SP, Brazil), naltrexone (Révia^®^, Cristália - Produtos Químicos Farmacêuticos Ltda., Itapira, SP, Brazil), and bupropion (Zyban^®^, GlaxoSmithKline Brasil Ltda., Rio de Janeiro, RJ, Brazil).

In the 12^th^ week of the experiment (end of the treatment) animals were weighed and euthanized by anesthetic overdose with sodium thiopental. After euthanasia a fragment of skin of animals was collected for analysis of subcutaneous adipose tissue located in the hypodermis.

### Microscopy of light

Organs were fixed in 10% formalin for 24 hours. Dehydrated in graded concentrations of alcohol, diaphanized, and embedded in paraffin. Sections of 7µm were made using a Leica RM-2245 microtome (Leica, Nussloch, Germany) and slides were stained with hematoxylin and eosin.

The slides were analyzed using a Nikon E200 microscope and afterwards, the slides were photographed using a Nikon Eclipse E800 photomicroscope and images were captured using the NIS- Elements 3.0^®^ image capture program.

### Morphometry

#### Estimation of adipose tissue volume density

To evaluate the volume density occupied by the subcutaneous adipose tissue it was necessary to use a reticle of dots superimposed on the photomicrography, as well as to perform the differential counting of the dots that focus on the adipose cells. According to Weibel,^(13)^ the sum of the partial volume fractions is equivalent to the unit volume that a given component occupies by means of the following relation:


$$V_{v}=\frac{P1}{P}$$

Where:

Vv= volume density of a given component;

P1= number of points incidents on the component under study;

P= total of points incident on the volume unit.

### Biochemical analysis

Blood samples were collected from animals via the abdominal aorta. For plasma separation, the blood was centrifuged at 1,500rpm for 10 minutes at room temperature. To obtain the concentration of the biochemical parameters glucose, total cholesterol and triglycerides, the calorimetric methodology was used and the BIO-2000 device (BIOPLUS) following the manufacturer’s recommendations.

### Statistical analysis

The study was based on mean and standard deviation for quantitative analyses. To estimate subcutaneous adipose tissue volume the Kruskal-Wallis test was performed followed by Dunn’s test. To analyze body weight and biochemical results, TWO-WAY ANOVA test was performed. Results with p value ≤0.05 were considered significant. All values were performed using GraphPad Prism 5 software.

## RESULTS

### Histological results

**Figure 1:**
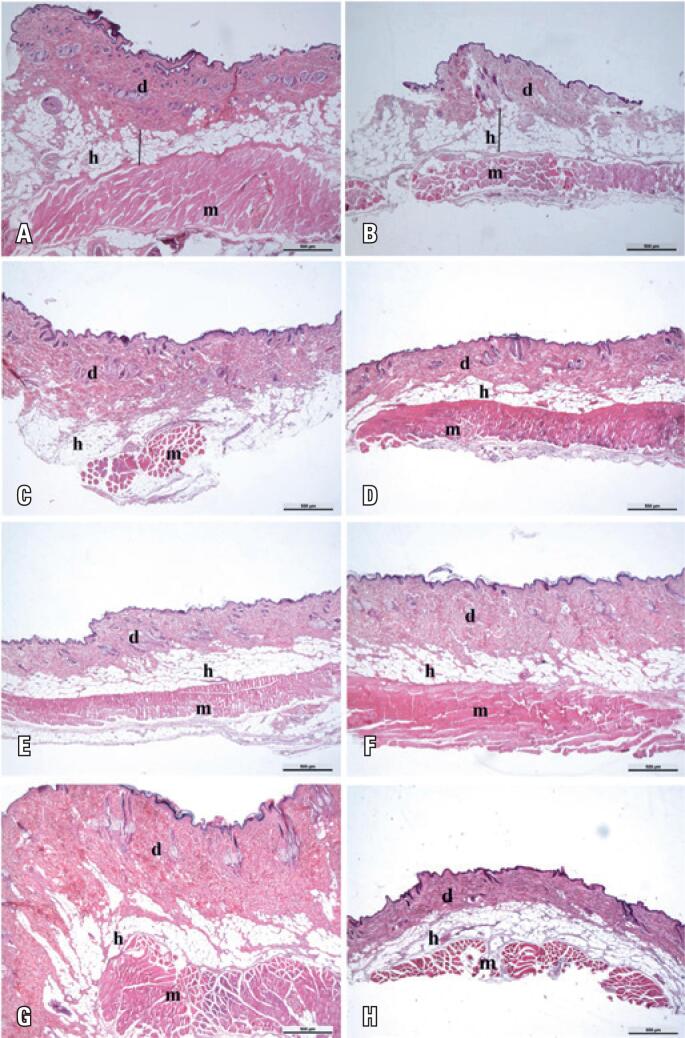
Photomicrograph of rat skin stained with hematoxylin and eosin. In (A), Control Group; (B) Group 0; (C) Group 1; (D) Group 2; (E) Group 3; (F) Group 4; (G) Group 5 and (H) Group 6. Note the regions of dermis, hypodermis, and muscle

HE: hematoxylin and eosin; d: dermis; h: hypodermis; m: muscular.

In the histological analyses, no morphological differences were found in the tissues that form the skin (epidermis and dermis) in the analyzed groups. Analyses made in the hypodermis, region where the subcutaneous adipose tissue is located, allowed us to observe that the hypodermis of the animals from the Control and G3 Groups presented in this region were smaller compared with the hypodermis of the animals from the other groups (Figure 1).

### Animal weight

Our results showed that from the first to the eighth week, the animals in all the studied groups, excepting the Control Group, had an increase of approximately 50% in their body weight (Table 1). Table 1 shows the mean weight of each group and the percentage (%) of weight gain. This percentage can be seen that from the first to the eighth week, the period without drug treatment, in which the animals in the groups increased their weight by more than 54%. From week 8 (start of drug treatment) to week 12 (end of treatment) the animals increased their weight by 9%. The exception is G3 that, with drug treatment, has its weight reduced by -0.4%.

**Table 1:** Percentage of weight gain

Weighteining week	No treatment		With treatment	
	1^st^ week	8^th^ week (%)	8^th^ week	12^th^ week (%)
Ctr	190.8g	250g (31)	250g	256.5g (2.6)
G0	182.4g	348.8g (50)	348.8g	364.4g (4.2)
G1	192.8g	375.6g (51.3)	375.6g	387.6g (3)
G2	203.6g	376.8g (54)	376.8g	386g (2.4)
G3	184.8g	376.8g (49)	376.8g	375g (-0.4)
G4	184g	370g (50)	370g	384g (3.7)
G5	209.6g	366.4g (57.2)	366.4g	401.6g (8.8)
G6	178.8g	360.8g (49.5)	360.8g	391.6g (7.9)

Ctr: Control Group; G0: Group 0; G1: Group 1; G2: Group 2; G3: Group 3; G4: Group 4; G5: Group 5; G6: Group 6.

**Figure 2:**
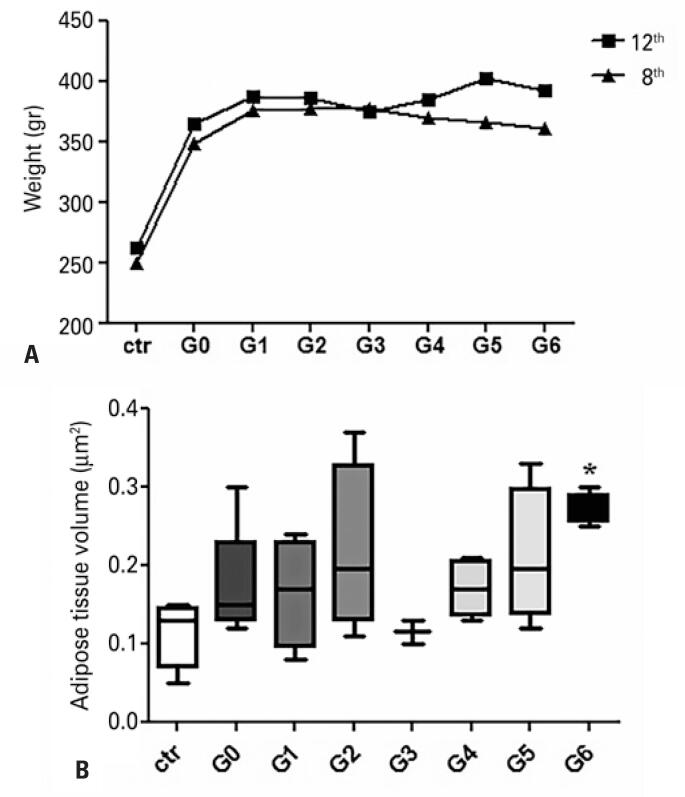
In (A) graph representing the weight means of the groups from week 8 (start of drug treatment) to week 12 (end of drug treatment). In (B) graph representing the means and standard deviations of adipose tissue volume

From the eighth week until the end of the drug treatment, our results showed that animals in Group G3 obtained a weight reduction of approximately 0.4%, however, all other groups studied had weight gain (Table 1) (Figure 2A).

The values were significant (p=0.05) where * represents the comparison of G6 with the Control Group. Ctr: Control Group; G0: Group 0; G1: Group 1; G2: Group 2; G3: Group 3; G4: Group 4; G5: Group 5; G6: Group 6.

### Estimation of adipose subcutaneous tissue volume

The amount of subcutaneous fat was significantly higher only in Group G6 (0.27±0.01*µ*m^2^) compared with the Control Group (0.11±0.04*µ*m^2^) (p=0.0481), Groups G0 (0.17±0.07*µ*m^2^), G1 (0.16±0.07*µ*m^2^), G2 (0.21±0.10*µ*m^2^), G4 (0.17±0.03*µ*m^2^) and G5 (0.21±0.08*µ*m^2^) that showed a greater proportion of subcutaneous adipose tissue than the animals in the Control Group. However, we did not find significant differences between them. Group 3 (0.11±0.02*µ*m^2^) presented the volume of adipose tissue similar to the Control Group (Figure 2B).

### Biochemical results

**Figure 3:**
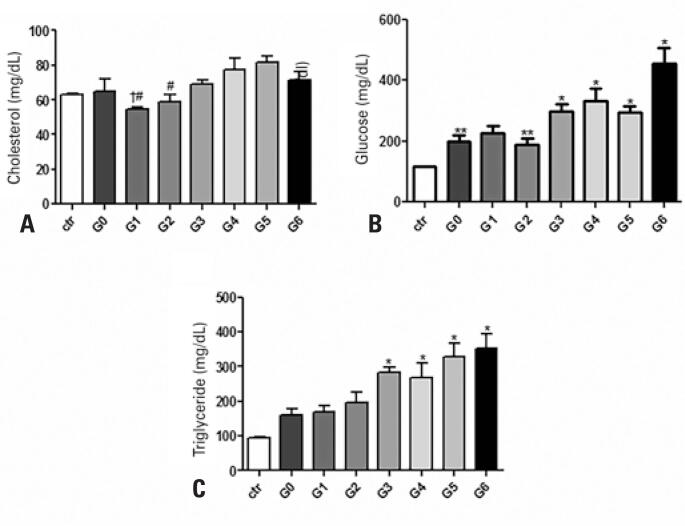
Biochemical tests. (A) The graph represents blood glucose concentration; (B) Represents cholesterol values and (C) Triglyceride values

Biochemical results show that amount of glucose in the blood was significantly higher in Groups G3 (298.9±57.6mg/dL), G4 (332.3±98.4mg/dL), G5 (293.3±52mg/dL) and G6 (545.6±131.8mg/dL) compared with Control Group (117.3±1.2mg/dL) (Figure 3A).

The value was significant (p=0.05) where * is *versus* Control Group; ** versus G6 Group; ^†^ *versus* G4 Group; ^#^ *versus* G5 Group. Ctr: Control Group; G0: Group 0; G1: Group 1; G2: Group 2; G3: Group 3; G4: Group 4; G5: Group 5; G6: Group 6.

The amount of cholesterol in the blood was homogeneous among the groups with the exception of G1 (54.3±4.1mg/dL), which showed a lower amount of cholesterol that was significant when compared with Groups G4 (77.2±16.1mg/dL) and G5 (81.7±9.3mg/dL) (Figure 3B).

The amount of triglycerides in blood was significantly higher in Groups G3 (282.6±41.5mg/dL), G4 (268.3±103.3mg/dL), G5 (328.7±96.7mg/dL) and G6 (352.3±104.6mg/dL) compared with the Control Group (94.7±3.6mg/dL) (Figure 3C).

## DISCUSSION

Our results show that the only group that presented body weight reduction was the group treated with naltrexone, however, this reduction was not significant. All animals treated with topiramate, bupropion, or a combination of these drugs gained weight.

Naltrexone is a drug initially approved and used as a treatment for opiate dependence, and later for alcohol dependence. The effect of this drug on weight loss may be related to its mechanism of action, as well as the use of Naltrexone that promotes the release of α-melacortin (α-MSH). The α-MSH is known to be an anorexigenic substance since it blocks the release of β-endorphins, which are substances commonly known to increase hunger. We did not find studies in the published scientific literature showing the effect of naltrexone in reducing body weight, but we believe that the weight loss of the rats in this group occurred due to the inhibition of β-endorphins promoted by the drug and for the consequent accumulation of α-MSH.

Bupropion is an aminocetone antidepressant that inhibits the reuptake of dopamine and noradrenaline.^(14)^ It is known that the excess of dopamine and noradrenaline in the synaptic cleft activates POMC receptors that, once activated, cleave into 2 peptides named α-MSH and β-endorphins. Since α-MSH is an anorexigenic substance, it promotes a decrease in body weight. However, this decrease in body weight occurs in the short term, since there will be a feedback effect of β-endorphins that inhibit the anorexigenic effect of α-MSH and activates hunger center of the brain.^(15)^ Our results are in agreement with the physiological effect of bupropion, since the animals gained weight over the treatment period.

Our result goes against most studies in the scientific literature. The fact that our results are not in agreement with most studies on the use of bupropion in weight loss can be justified, since these studies were conducted in humans and used bupropion treatment associated with a low-calorie diet, with different body mass index ranges, *i.e.*, the weight loss effect proposed by these studies might be associated with diet and not only with the use of the drug.^(16)^

Topiramate is initially a drug for the treatment of epilepsy and extended to treat migraine,^(9)^ but it has been shown to be effective in reducing weight in obese individuals with binge eating disorder.^(10)^ The effect of topiramate on weight reduction is unknown, however, in scientific study models with obese animals, topiramate seems to reduce appetite and, consequently, promote weight reduction.^(9)^ Our results are contrary to the finding reported in the literature, because animals treated with topiramate did not show weight reduction; on the contrary, weight gain of approximately 3.7% was observed.

The association of topiramate with bupropion is quite interesting since both increase energy expenditure and decrease appetite. We did not find studies that prove the effectiveness of these 2 drugs associated in weight loss, however, our results showed that the animals that took the 2 drugs together gained approximately 2.4% weight.

The association of the 3 drugs (naltrexone, bupropion, and topiramate) has been widely used in clinical practice as for weight loss protocol. However, there are no clinical and experimental studies in the literature that show the effect of these compounds together. In our results, the association of the 3 drugs was not effective in the weight loss of the animals, since they presented a 7.9% increase in weight.

Our results showed that only naltrexone was effective in reducing weight. Comparing the results of the weight of the animals and the volume of subcutaneous adipose tissue, we observed that the 2 data are in agreement, a fact that supports the quality of this study.

Our results were contrary to most studies in the published literature, and this may be attributed to the treatment time used in our study, which was only 30 days. We suggest that future studies should study the effects on weight loss of these drugs associated for more than 30 days. Clinical practice has shown excellent results in the long-term use of these drugs, associated or not, in the treatment of BED and, consequently, in the weight loss process in humans. Considering that this study was conducted in rats and with a forced feeding through gavage method, we believe that studies involving the analysis of the feeding behavior of the animals, particularly those treated with the studied drugs, may be important to prove the effect of these drugs on weight loss.

A fact to be considered in this study were the biochemical results, since the highest values of glucose and triglycerides were observed in the treated groups. We attributed the increase in these organic compounds to the weight gain that was observed in the animals. This claim can be established particularly for the endorsemtn in the published literature that overweight animals present increased concentrations of glucose and triglycerides.

## CONCLUSION

All the drugs studied were not effective in reducing weight, excepting the naltrexone. A longer period of treatment should be considered in order to observe significant changes.
